# A New Model to Describe the Single-dose Pharmacokinetics of Bevacizumab and Predict Its Multiple-Dose Pharmacokinetics in Beagle Dogs

**DOI:** 10.22037/ijpr.2019.1100716

**Published:** 2019

**Authors:** Meizhen Li, Wei Qiang, Zhou Wen, Linling Li, Lei Wang, Zeneng Cheng

**Affiliations:** a *Research Institute of Drug Metabolism and Pharmacokinetics, School of Pharmaceutical Sciences, Central South University, Changsha, Hunan 410013, China.*; b *Department of Cell Biology, School of Life Sciences, Central South University, Changsha, Hunan 410013, China.*; 1 M. L. and W. Q. contributed equally to this work.

**Keywords:** Elimination, Metabolism, Modeling, Monoclonal antibodies, Simulations

## Abstract

Complex pharmacokinetic (PK) properties including nonlinear elimination were encountered by some monoclonal antibodies (mAbs), and classic compartment models sometimes failed to appropriately describe those properties. In this work, a new model was built on a comprehensive analysis of the complex elimination of mAbs. This new model was firstly utilized to fit with the single-dose plasma concentration data of bevacizumab in beagle dogs receiving an intravenous administration of 2.5 mg/kg bevacizumab. Then, the optimal PK parameters from fitting with the single-dose PK data were employed into the multiple-dose mathematical expressions to predict bevacizumab’s multiple-dose PK profiles. One-compartment model recommended as the optimal classic model by DAS 2.0 software was set as a control. As a result, new model fitted better with the single-dose PK profiles of bevacizumab with smaller weighted residual sum of squares and higher fitting degree compared with the classic model. Importantly, new model also accurately predicted the multiple-dose PK profiles of bevacizumab and performed well at the single-to-multiple transition. In conclusion, the new model reasonably explained the complex elimination of bevacizumab, and it might play a big role in the PK studies of bevacizumab and other mAbs.

## Introduction

Monoclonal antibodies (mAbs) offer considerable advantages over small-molecule drugs through a specific bind to target antigens relevant to diseases progress, by increasing the efficacy of treatment and/or by having fewer adverse effects than conventional therapy (1, 2). Bevacizumab, a recombinant humanized IgG1 antibody, has been widely used for the treatment of metastatic colorectal cancer and non–small cell lung cancer as a kind of anti-angiogenic agent by inhibiting effects of vascular endothelial growth factor (VEGF) (3, 4).

Pharmacokinetic (PK) analyses play an important role in the drug discovery and development process (5, 6). Particularly in early clinical trials, PK study is helpful for the selection of doses in further trials and promotion of clinical rational drug use (7, 8). Regarding the PK analyses of small-molecule drugs, classic compartment models are widely used for charactering drugs’ PK properties with model parameters, such as half life, maximum concentration (C_max_), clearance, and so on (9, 10). Classic compartment models are built on an assumption that the elimination of drugs is a first-order rate process. However, nonlinear elimination is sometimes encountered by some mAbs, and classic compartment models used for linear elimination may fail to make an accurate description of the PK of those drugs (11, 12). 

Some new models have been proposed to describe PK profiles of mAbs in recent years (11, 13 and 14). The target-mediated drug disposition (TMDD) model, built by Mager’s group, analyzes the kinetics of targets, antibodies, and target-antibody complexes respectively, and then integrates the PK of antibodies with those of targets and target-antibody complexes. TMDD model fits well with the PK profiles of some mAbs due to a successful description of the interaction between antibodies and their targets (13, 14). However, TMDD model consists of numerous parameters and the value of some parameters are only available through other complicated experiments such as fluorescence-activated cell sorting analysis and so on, thus probably limiting its extensive applications (11).

It has been well known that mAbs are significantly different with small-molecule drugs not only in the pharmacological mechanism but also in the elimination mechanism (12, 15 and 16). Regarding mAbs, renal elimination is relatively unimportant as its large size prevents efficient filtration through the glomerulus. The elimination of mAbs is complicated. They will encounter a target-mediated endocytosis as endogenous IgG triggered by binding to its targets such as antigens, receptors, and some proteins or polypeptides (16-18). Meanwhile, the degradation and metabolism of protein and/or a phagocytosis of lymphocyte further complicate the elimination of mAbs (15, 19).

In this work, a new model was built on the complex elimination of mAbs, which consisted of a zero-order rate process and a first-order rate process. The proposed model was used for describing the single-dose PK of bevacizumab in beagle dogs, and then it was utilized to predict multiple-dose PK profiles by employing the parameters derived from fitting with the single-dose PK data. 

## Experimental

*New PK Model*

**Figure 1:**
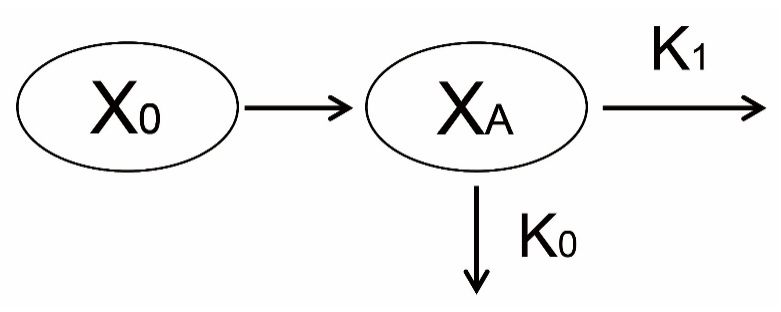
Model diagram of the new model describing the PK of mAbs in beagle dogs

The model diagram was illustrated in Figure 1. At the first administration of mAbs, the concentration of mAbs decreased firstly because mAbs bound with pre-existing targets. Then, the rest of mAbs were eliminated mainly through two pathways. On the one hand, due to the nature of proteins or peptides, mAbs encountered a metabolism in an analogous way as most kinds of proteins or peptides did *in-vivo* (15, 19). The apparent elimination rate of this pathway was assumed to be first-order. On the other hand, binding with newly generated endogenous targets would trigger antibody dependent cellular cytotoxicity (ADCC) and/or complement activity to eliminate mAbs from bodies (16-18). The rate of this pathway was assumed to be zero-order. A superposition of a first-order process and another zero-order process complicated the elimination of mAbs *in-vivo*.

As administrated intravenously, mAbs (X_0_) firstly went through an initial elimination to decrease its concentration by binding with pre-existing targets. Subsequently, the rest of mAbs (X_A_) underwent a complex elimination consisting of one first-order process (its rate constant was K_1_) and another zero-order process (its rate constant was K_0_).

The PK models were mathematically described with the following Equations:

$C=C_{A}.e^{{-k}_{1}t}-\frac{K_{0}}{K_{1}V}(1-e^{{-k}_{1}t})$

(1)

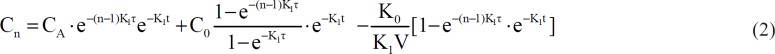


Equation 1 is applied to describe the single-dose PK process with an intravenous administration, and Equation 2 is for the description of the multiple-dose PK profile accordingly. C_A_ represents the maximum drug plasma concentration after a prompt bind with pre-existed targets at the first administration; C_0_ represents the initial plasma concentration after each administration in multiple-dose trials; K_1_ represents the first-order elimination rate constant of mAbs, and it describes the kinetics of the process where mAbs eliminate in an analogous way as most kinds of proteins or peptides; K_0_ represents the zero-order elimination rate constant, describing the kinetics of the pathway where mAbs eliminate through binding with newly generated target; τ is dosing interval; V represents the volume of 

distribution. 

**Figure 2:**
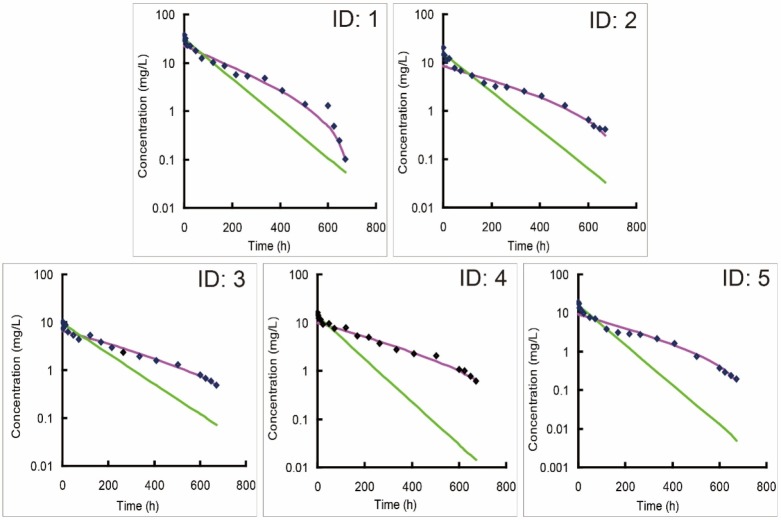
Individual predicted versus observed concentration-time profiles of bevacizumab in five beagle dogs (ID: 1-5) following an intravenous administration of 2.5 mg/kg bevacizumab. New model (purple line) was utilized to fit with observed concentration data, and one-compartment model (green line) was recommended as the optimal classic model by DAS 2.0 software to fit those data for a comparison

**Figure 3:**
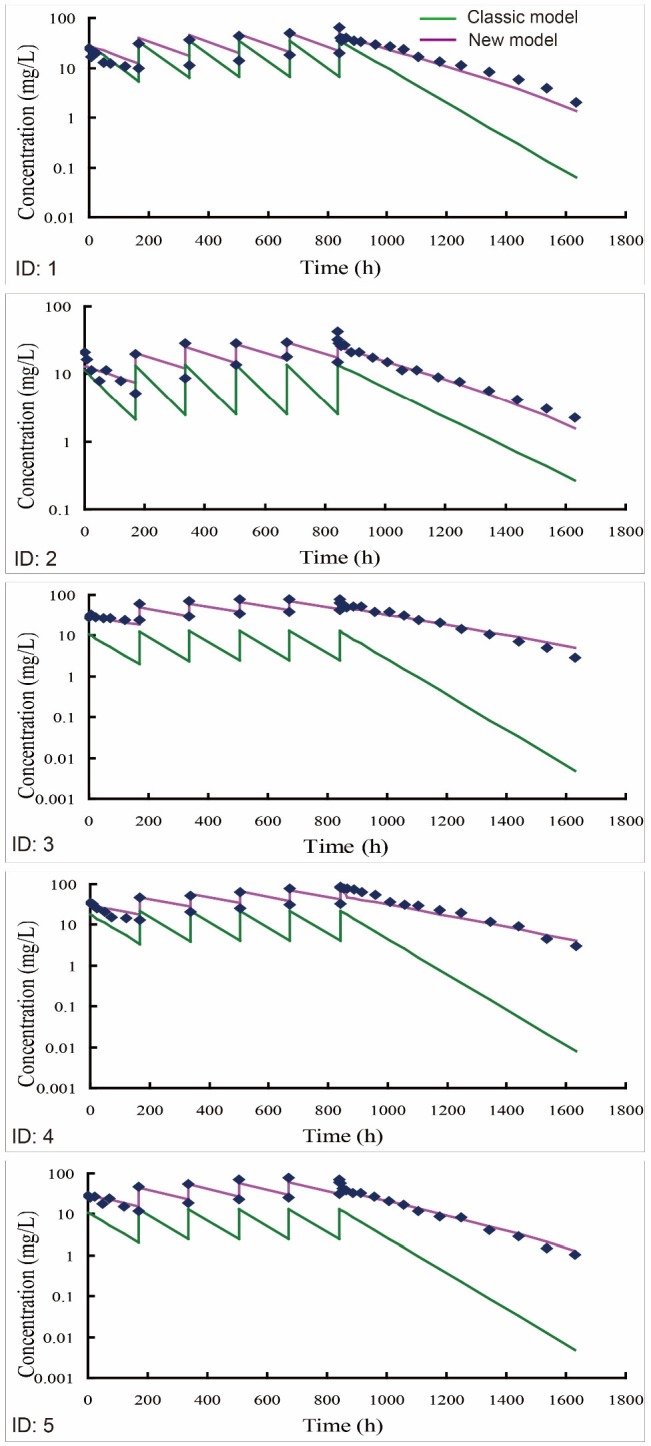
Individual predicted versus individual observed concentration-time profiles of bevacizumab in 5 beagle dogs (ID: 1-5) receiving an intravenous infusion of 2.5 mg/kg bevacizumab once a week for six weeks. One-compartment model was recommended as the optimal classic model by DAS 2.0 software. The multiple-dose PK profiles of bevacizumab were simulated using either new (purple line) or classic model (green line) by employing the PK parameters from fitting with the single-dose PK data

**Table 1:** Weighted residual sum of squares and fitting degree of two models fitting with individual single-dose PK data

**ID**	**Weighted residual sum of squares**		Fitting Degree	
	**Classic compartment model**	**New model**	**Classic compartment model**	**New model**
1	176.32	3.70	0.957	0.983
2	69.71	0.50	0.894	0.989
3	36.14	0.35	0.948	0.983
4	144.44	0.46	0.864	0.987
5	60.07	1.33	0.897	0.959

**Table 2:** Parameters of new model by fitting with the single-dose PK data of bevacizumab

**ID**	**K** **1 ** **(1/h)**	**C** **A ** **(mg/L)**	**K** **0** **/V (mg/h × L)**
1	0.0048	22.55	0.0058
2	0.0030	8.45	0.0032
3	0.0028	6.65	0.0045
4	0.0030	9.84	0.0039
5	0.0039	9.72	0.0072

**Table 3:** Prediction of the steady state plasma drug concentration with new model after multiple-dose administrations in beagle dogs

**ID**	**C** **min** **ss (mg/L)**			**C** **max** **ss (mg/L)**		
	**C-obs** **a**	**C-pre** **b**	**RE (%)** **c**	**C-obs**	**C-pre**	**RE (%)**
1	19.75	21.37	8.2	64.74	48.95	24.3
2	15.20	17.38	14.3	32.48	30.37	6.5
3	42.17	44.63	5.8	79.56	74.61	6.2
4	32.44	41.39	27.6	84.49	71.37	15.5
5	31.15	30.67	1.6	70.73	60.64	14.3

aC-obs represented the observed concentration, it obtained from a detection of the plasma samples collected in the multiple-dose trial. bC-pre stood for the prediction concentration, which derived from a stimulation with new model by employing the single-dose PK parameters into the multiple-dose mathematical expressions.

cRE represented relative error, which was accumulated as RE = (C-pre - C-obs)/C-obs.

**Table 4:** Predictions of the multiple-dose pharmacokinetic profiles with new and classic compartment models

**ID**	**Weighted residual sum of squares**		**Fitting degree**	
	**Classic compartment model**	**New model**	**Classic compartment model**	**New model**
1	745.63	37.26	0.856	0.960
2	607.28	17.55	0.669	0.972
3	15541.34	26.20	0.300	0.979
4	9890.69	95.83	0.437	0.944
5	4510.80	21.27	0.378	0.976

*Chemical and Reagents*

Bevacizumab (Avastin, 100 mg/4 mL) was purchased from the manufacturer (Genentech, CA, USA). Recombinant human VEGF_165­ _(Peprotech, USA) was immobilized on solid phase surface of ninety-six-well plates (Greiner, Germany) to capture bevacizumab. Five percent nonfat dried milk (Dingguo Changsheng Biotechnology, China) dissolved in phosphate-buffered saline (PBS) (Dingguo Changsheng Biotechnology, China) was used to seal the solid phase surface of each well, and 0.5% Tween-20 (Damao Chemical Reagent Factory, China) in PBS worked as a wash solution. Horseradish peroxidase-goat anti-human IgG (H+L) conjugate (ABclonal Technology, UK) was employed to detect bevacizumab. Tetramethyl benzidine (TMB) (Solarbio, China) and 2 mol/L hydrogen chloride (Sinopharm Chemical Reagent, China) were prepared in the laboratory to work as substrate solution and stop solution, respectively. 

*PK Study in Beagle Dogs*

The animal studies were approved by the Animal Ethics Committee of the Third Xiangya Hospital of Central South University. All experiments were conducted in accordance with the National Institute of Health Guide for the Care and Use of Laboratory Animals. 

Five beagle dogs with three males and two females, weighing 7.1 to 9.2 kg, were purchased from Rixin Technology Co., Ltd. (Beijing, China). All beagle dogs were placed in individual stainless steel metabolism cages and provided with a 12 h light-dark cycle at an ambient temperature of 21–22 °C. Animals received standard laboratory dog diet and water. 

In the single-dose PK trial, all dogs received an intravenous infusion treatment of 2.5 mg/kg of bevacizumab. Two mL blood was collected from the foreleg vein into heparinized centrifuge tubes before and at 0.083, 2, 6, 12, 24, 48, 72, 120, 168, 216, 264, 336, 408, 480, 504, 600, 624, 648, and 672 h after administration.

After a one-month washout, five dogs received repeated dose of bevacizumab of 2.5 mg/kg once a week. Two mL blood was collected from the foreleg vein into heparinized centrifuge tubes as following scheme: 1) before, and at 0.083, 6, 24, 48, 72, and 120 h postdose at the first administration; 2) before, and at 0.083 h postdose from 2^nd^ to 5^th ^administration; 3) before, and at 0.083, 2, 6, 12, 24, 48, 72, 120, 168, 216, 264, 336, 408, 504, 600, 696, and 792 h postdose at the last administration. 

The blood samples were immediately centrifuged at 440×g for 10 min after collection, and then the plasma was separated and stored at -20 °C until assay.

*Assay Method*

The concentration of bevacizumab was measured with an enzyme-linked immunosorbent assay as previously described with slight modification (20). Firstly, 1 μg/mL recombinant human VEGF_165­_ were coated on solid phase surface of ninety-six-well plates (100 μL/well), and then they were incubated overnight at 4 °C. Secondly, washing the wells three times with phosphate-buffered saline containing 0.05% Tween-20 (PBST), and blocking them with 5% nonfat dried milk/PBST (200 μL/well) by incubating at 37 °C for 2 h. Thirdly, after removing block solution, plasma sample diluted in 1% nonfat dried milk/PBST was added to the plates (50 μL/well) with an incubation at 37 °C for 1 h. Fourthly, washing the wells five times with PBST, and then detecting bevacizumab with 1 μg/mL horseradish peroxidase goat anti-human IgG (H+L) conjugate after incubating at 37 °C for l h. Finally, after the wells were washed five times with PBST, color development was performed with an addition of 100 μL tetramethyl benzidine substrates (3, 3’, 5, 5’-tetramethyl benzidine substrate) into each well, and the reaction was subsequently stopped with 1 mol/L sulfonic acid (100 μL/well). The optical density was determined at 450 nm with correction wavelength set at 570 nm. This assay measured the concentration of free bevacizumab. A standard curve ranging from 25 to 800 pg/mL was obtained, and the concentration in each sample was measured twice.

*Modeling and Prediction*

The single-dose PK data of bevacizumab was fitted by either new or classic compartment models using Matlab 7.0 (MathWorks, USA) and DAS 2.0 software respectively, and weighted residual sum of squares and fitting degree were calculated to evaluate the accuracy of model fitting of models by setting the reciprocal of model-predicted concentration as the weighted factor. Multiple-dose PK profiles were simulated using either new or classic compartment models, by employing the PK parameters derived from fitting with single-dose PK data. In the simulation, the concentration of bevacizumab at each collected time was calculated with either new or classic model, and then the predicted value was compared with the real value which were obtained by detecting the plasma samples. The relative errors (REs%) were calculated to evaluate the accuracy of the simulations of both models when fitting the bevacizumab PK data in multiple-dose PK study, using the following Equation:

$\mathrm{RE\%}=[\frac{\mathrm{predicted concentration}-\mathrm{observed concentration}}{\mathrm{observed concentration}}]\times 100$

(3)

Meanwhile, the weighted residual sum of squares and fitting degree were calculated to evaluate the fitting accuracy of both models by setting the reciprocal of the model-predicted concentration as the weighted factor. 

## Results

*Modeling of single-dose PK data using either new or classic compartment models*

One compartmental model was recommended as the optimal model by DAS 2.0 software when modeling of the single-dose PK data of bevacizumab with classic compartment models. The individual simulations of new model matched better against the individual observations than classic models (Figure 2), suggesting that new model fitted better with the single-dose PK profiles of bevacizumab compared with classic models. Accordingly, the weighted residual sum of square of the modeling by new model was decreased and its fitting degree was also improved compared with those by classic models (Table 1). It also proved the stronger ability of new model in terms of describing the single-dose PK profiles of bevacizumab in beagle dogs. Parameters from fitting with bevacizumab single-dose PK data with new model were also listed in Table 2.

*Predictions of multiple-dose PK profiles using either new or classic compartment models*

Predictions of multiple-dose PK profiles (2.5 mg/kg) were further performed by employing the optimal parameters from single-dose PK data into the multiple-dose mathematical expressions. The model predicted plotted against observed data was illustrated in Figure 3. The individual predicted of new model matched well against the individual observations, namely new model accurately predicted the multiple-dose PK profiles of bevacizumab in beagle dogs. On the contrary, the predictions of classic models were obviously lower than the individual observations, indicating that the classic models underestimated the observed PK profiles. 

In addition, regarding the steady state of multiple dosing, both minimum plasma concentration (C_min_ss) and maximum plasma concentration (C_max_ss) were generally predicted within 20% observations (Table 3), implying that new model successfully predicted the steady states after multiple-dose administration. Moreover, as listed in Table 4, new model exhibited an improved fitting capacity for the multiple-dose PK data compared with classic models with smaller weighted residual sum of squares and higher fitting degree.

## Discussion

Classic compartment models are built on the assumption that the elimination of drug is a first-order process *in-vivo*, and its rate is related to drug’s concentration, which represents a linear profile in the logarithmic concentration-time curve (21). For most drugs, especially small-molecule drugs, their eliminations comply with this first-order rule, thus classic models can fit well with their PK profiles. However, bevacizumab displayed a nonlinear elimination profile in the single-dose PK trial, and the classic models were hard to fit well with those profiles, especially fitted unsatisfactorily with the data in the elimination phase (Figure 2). It indicated that the elimination of bevacizumab might not be a simple first-order process. 

New model is built on that the apparent elimination of mAbs is a superposition of a first-order process and a zero-order process. As the concentration of drugs is in a relative high level, its first-order elimination, whose rate is related to drug’s concentration, is so fast that it covers the zero-order elimination. In the case, the apparent elimination seems to be a first-order process. However, as the concentration decreasing, the rate of first-order elimination slows down while the rate of zero-order elimination is constant, thereby causing a continued variation of the ratio between first-order elimination and zero-order elimination. Accordingly, the apparent elimination deviates from the original first-order process, and an inflexion appears in the concentration-time profile. On the basis of the hypothesis, new model has made a reasonable explanation about the nonlinear elimination profile of bevacizumab, proving that bevacizumab underwent a complex elimination rather than simple first-order elimination *in-vivo*.

Repeated administration is required for the treatment of some diseases in clinical practice. A robust performance at the single-to-multiple transition is essential for a reliable PK model. For the classic compartment models, its individual predictions were obviously lower than the observations (Figure 3). The elimination of bevacizumab was overestimated by classic models for two reasons. Firstly, the concentration of bevacizumab decreased initially because of bevacizumab binding with the pre-existing VEGF at the first administration. The initial elimination process caused by the pre-existing targets, however, was automatically treated as a part of the first-order elimination in the model fitting by classic models, causing an overestimation of its elimination rate. Secondly, though pre-existing VEGF imposed an influence on the PK process of bevacizumab by triggering an initial elimination at the first administration, its effect on the subsequent dosing ought to be removed because pre-existing VEGF had been neutralized in the initial elimination. However, the initial elimination was viewed as a default process and its effect was counted repeatedly at each dosing in the predictions by classic models. This magnified the contribution of initial elimination to the apparent elimination of bevacizumab. For above two reasons, the elimination of bevacizumab was overestimated by classic models eventually.

On the contrary, new model had isolated the initial elimination from the apparent elimination, and the effect of pre-existing targets on the multiple-dose PK had been fully considered as well. New model had evaluated the effects of different parts of eliminations respectively, rather than treated them equally as one first-order process like what classic models did. Moreover, the effects of initial elimination caused by mAbs binding with pre-existing targets was not counted repeatedly but removed at the second dosing when simulating the multiple-dose PK profiles by the new model. Therefore, new model finally showed better performance at the single-to-multiple transition.

An accurate prediction of multiple-dose PK profiles plays an important role in the personalized medicine and dose adjustment in clinic. Through obtaining the personalized parameters by fitting with single-dose PK data, an appropriate dosage regimen is to build to keep drug’s concentration at a level that exactly neutralizes pre-existing targets and eliminates new targets. In addition, it will help us to promptly adjust the dose and/or the dosing interval to locate the steady state plasma drug concentration into the therapeutic window, by making predictions of the multiple-dose PK profiles. 

Although the new model was only utilized to fit with bevacizumab PK data in beagle dogs, it offered us a new idea to describe the complex PK profiles of mAbs. Each mAb is specific to one kind of disease-related target, due to a specific connection between their antigen-binding fragments and the complementary structures of targets just like one key applied to one lock (22). When the target is soluble in body fluid or bound in cell surface, its binding with according mAb will trigger ADCC and/or complement activity to induce the zero-order elimination of mAbs (16-18). In addition, the first-order elimination of mAbs derived from a general metabolism as its nature of proteins or peptides, and this part of elimination will be similar among different mAbs (19). For these reasons, a similar complex elimination should be encountered by other mAbs, and new model may also be available for the modeling of those mAbs.

## Conclusion

In summary, new model well described the PK profiles of bevacizumab based on a reasonable explanation of its complex elimination. Importantly, the proposed model performed well at the single-to-multiple transition as well. Although some details of the complex elimination mechanism are required to be clarified in further studies, this new model may be an appropriate tool for the PK studies of bevacizumab and other mAbs in practice.
